# Toward Elimination of Dog-Mediated Human Rabies: Experiences from Implementing a Large-scale Demonstration Project in Southern Tanzania

**DOI:** 10.3389/fvets.2017.00021

**Published:** 2017-03-06

**Authors:** Emmanuel Abraham Mpolya, Tiziana Lembo, Kennedy Lushasi, Rebecca Mancy, Eberhard M. Mbunda, Selemani Makungu, Matthew Maziku, Lwitiko Sikana, Gurdeep Jaswant, Sunny Townsend, François-Xavier Meslin, Bernadette Abela-Ridder, Chanasa Ngeleja, Joel Changalucha, Zacharia Mtema, Maganga Sambo, Geofrey Mchau, Kristyna Rysava, Alphoncina Nanai, Rudovick Kazwala, Sarah Cleaveland, Katie Hampson

**Affiliations:** ^1^Global Health and Biomedical Sciences, School of Life Sciences and Bioengineering, Nelson Mandela African Institution of Science and Technology, Arusha, Tanzania; ^2^Institute of Biodiversity, Animal Health and Comparative Medicine, University of Glasgow, Glasgow, UK; ^3^Paul G. Allen School for Global Animal Health, Washington State University, Pullman, WA, USA; ^4^Ifakara Health Institute, Dar es Salaam, Tanzania; ^5^Department of Epidemiology, Ministry of Agriculture, Livestock and Fisheries, Dar es Salaam, Tanzania; ^6^Preventive Veterinary Medicine, Sokoine University of Agriculture (SUA), Morogoro, Tanzania; ^7^Food Safety Zoonoses and Food-Borne Diseases, World Health Organization (former WO staff), Geneva, Switzerland; ^8^Neglected Zoonotic Diseases, WHO, Geneva, Switzerland; ^9^Tanzania Veterinary Laboratory Agency (TVLA), Ministry of Agriculture, Livestock and Fisheries, Dar es Salaam, Tanzania; ^10^Department of Epidemiology, Ministry of Health, Community Development, Gender, Elderly and Children (MoHCDGEC), Dar es Salaam, Tanzania; ^11^Department of Neglected Tropical Diseases, World Health Organization – Country Office of Tanzania, Dar es Salaam, Tanzania

**Keywords:** Rabies Elimination Demonstration Project, mass dog vaccination, Southeastern Tanzania, One Health, SARE

## Abstract

A Rabies Elimination Demonstration Project was implemented in Tanzania from 2010 through to 2015, bringing together government ministries from the health and veterinary sectors, the World Health Organization, and national and international research institutions. Detailed data on mass dog vaccination campaigns, bite exposures, use of post-exposure prophylaxis (PEP), and human rabies deaths were collected throughout the project duration and project areas. Despite no previous experience in dog vaccination within the project areas, district veterinary officers were able to implement district-wide vaccination campaigns that, for most part, progressively increased the numbers of dogs vaccinated with each phase of the project. Bite exposures declined, particularly in the southernmost districts with the smallest dog populations, and health workers successfully transitioned from primarily intramuscular administration of PEP to intradermal administration, resulting in major cost savings. However, even with improved PEP provision, vaccine shortages still occurred in some districts. In laboratory diagnosis, there were several logistical challenges in sample handling and submission but compared to the situation before the project started, there was a moderate increase in the number of laboratory samples submitted and tested for rabies in the project areas with a decrease in the proportion of rabies-positive samples over time. The project had a major impact on public health policy and practice with the formation of a One Health Coordination Unit at the Prime Minister’s Office and development of the Tanzania National Rabies Control Strategy, which lays a roadmap for elimination of rabies in Tanzania by 2030 by following the Stepwise Approach towards Rabies Elimination (SARE). Overall, the project generated many important lessons relevant to rabies prevention and control in particular and disease surveillance in general. Lessons include the need for (1) a specific unit in the government for managing disease surveillance; (2) application of innovative data collection and management approaches such as the use of mobile phones; (3) close cooperation and effective communication among all key sectors and stakeholders; and (4) flexible and adaptive programs that can incorporate new information to improve their delivery, and overcome challenges of logistics and procurement.

## Background

Rabies is one of the oldest known zoonosis, which is defined as an acute progressive encephalitis that almost inevitably results in death without timely intervention (1). Rabies can be transmitted by several hosts, but domestic dogs are the main species that transmit the disease to humans (2). With a growing recognition of the need for One Health approaches to tackle zoonotic diseases (3), it has been recognized that a paradigm shift is required to tackle human rabies in low- and middle-income countries (LMICs), by focusing on immunization of the primary reservoir hosts, the domestic dogs.

In terms of the disease burden, rabies is responsible for an estimated 59,000 human deaths globally, about 96.0% of which occur in Africa and Asia (4). Particularly, Asia contributes 59.6% of annual deaths due to rabies, while Africa contributes 36.4% of annual deaths (4). The escalation of dog rabies across much of Asia and Africa is mainly due to the low priority given to control of the disease. This low priority is in turn due to a lack of awareness of the true scale and magnitude of the disease burden as well as misperceptions as to the feasibility, cost-effectiveness, and public health benefits of dog rabies control (5, 6).

Since most LMICs still focus on post-exposure prophylaxis (PEP) as the only means to prevent human deaths from rabies (4), there is a clear need for greater focus on dog vaccination as a more sustainable and cost-effective way of addressing rabies in humans (7–9). Within this context, a demonstration project in Tanzania was initiated to prevent human rabies through the control and eventual elimination of canine rabies while improving the delivery of PEP to exposed patients, as well as surveillance and diagnostics. Finally, the project aimed to build a strategy to ensure sustainability of the rabies-free status beyond the project duration as illustrated in the Stepwise Approach towards Rabies Elimination—SARE (10).

Critical milestones of the project were therefore the progressive reduction and eventual elimination of human deaths due to dog rabies together with decreased numbers of PEP doses delivered. These should be concomitant with a reduction of dog rabies cases and/or positivity rate of dog samples tested in the laboratory, coupled with increasing immunization coverage in the dog population and evidence of rabies-free status. In light of these milestones, we present our experiences of implementing this large-scale rabies control project.

## Methodology

### Project Areas

The project site in Tanzania included the mainland regions of Dar es Salaam, Lindi, Mtwara, Morogoro, and Pwani, as well as Pemba Island. This covered a total of 28 districts including 4 from Pemba, and 459 wards, as of the year 2010, with more than 8 million inhabitants. Initially, the area was estimated to have about 400,000 dogs, based on dog-to-human ratios generated from other areas of Tanzania extrapolated to the districts in Southern Tanzania with similar population characteristics (11). Through household and post-vaccination surveys, the actual number of dogs was subsequently determined to be between 100,000 and 150,000 with much more variable human-to-dog ratios within districts.

**Figure 1:**
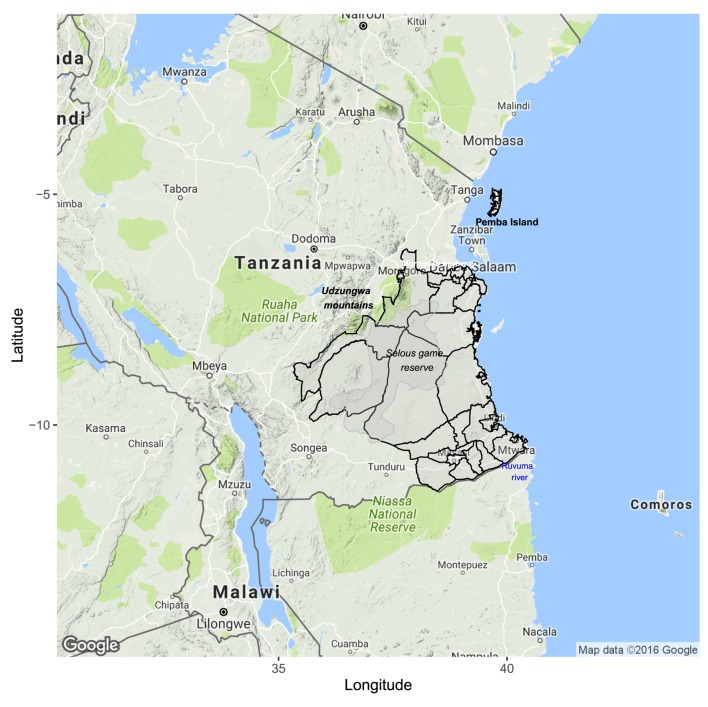
**Project area consisting of 28 districts from mainland Tanzania and Pemba Island**. Districts within the project area are demarcated in black with natural boundaries highlighted including the highway and railway in the north, the coastline in the east, mountains to the west, and the Ruvuma River in the south. The Selous Game Reserve is shaded in gray.

The area of implementation was selected to exploit natural boundaries to facilitate the establishment and maintenance of a rabies-free area, including the coastline to the east, Udzungwa Mountains to the northwest, and Ruvuma River to the south. The Dar es Salaam–Mbeya highway to Morogoro and railway line to Kilosa defined the northern boundary of the project zone (Figure 1).

### Project Management

The project was implemented collaboratively and primarily by bodies relevant to the animal and human health sectors with other sectors having a secondary role. The project organizational structure involved a national coordinator based at the World Health Organization (WHO)—Country Office of Tanzania. The National Coordinator worked with a multisector steering advisory committee, which consisted of representatives from key Tanzanian ministries dealing with livestock and human health through their focal persons, the WHO, the Sokoine University of Agriculture (SUA), and the University of Glasgow. Two laboratories dealt with diagnostic tasks: the Tanzania Veterinary Laboratory Agency (TVLA) in Dar es Salaam and the Sokoine University of Agriculture’s (SUA) Faculty of Veterinary Medicine Laboratory in Morogoro.

### Project Launch and Implementation

The project was internationally launched in 2009 when there was no large-scale rabies control program in Tanzania. In 2010, the project was initiated officially in Tanzania. Between 2009 and 2010, the necessary human and material resources for the project were set up, including infrastructure for implementation and working relationships between relevant sectors. With the aim to revaccinate each district annually, training sessions were delivered to district veterinary officers, district medical officers, health workers, and laboratory staff. In addition, standard operating procedures were established for control, prevention, and surveillance activities. Health information relevant to rabies was continuously delivered to community leaders, students, teachers, and the general public throughout the project.

Mass dog vaccination campaigns were conducted in phases, according to logistic constraints. Initially, the vaccination project focused on urban areas and then expanded to the entire project area, aiming to revaccinate each district annually. Household surveys and post-vaccination transects were used at different times during the project to estimate dog populations and vaccination coverage. Training and materials for sample collection and for diagnostic capacity were also provided by the project to district veterinary officers and livestock field officers. Fluorescent microscopes at the two laboratories were also refurbished with the support of the US Centers for Disease Control.

Under current national policy in Tanzania, PEP is distributed only to district hospitals. However, for this project, decentralized provision of PEP was established through training of staff and distribution of vaccines to four additional health facilities in each district. Health-care workers were trained in the more immunogenic and cost-effective intradermal (ID) administration of vaccine to animal bite victims (12) and in the use of human rabies immunoglobulins (RIG). PEP was provided free of charge to bite victims across the project areas.

### Data Collection

The Ministry of Health, Community Development, Gender, Elderly and Children (MoHCDGEC) routinely records animal bites and human rabies deaths throughout Tanzania and numbers of human vaccinations administered and distributed within each district. These data were initially compiled before the project began to assess vaccine needs. During the project, the Ministry of Agriculture, Livestock and Fisheries (MALF) collected data on dog vaccination, and estimated dog populations and vaccination administration costs (13).

From 2011 onward, a mobile phone-based surveillance system was established to collect more detailed information needed for evaluation of project progress (14). Phones were distributed to the four health facilities responsible for district-level provision of PEP and to the district livestock officers. Livestock field officers and health workers were trained on how to report using mobile phones, enabling more rapid collection of extensive data than routine paper-based approaches. Variables collected included animal bite patient records, human rabies deaths (based on clinical criteria), PEP doses (demand and shortages), animals vaccinated during village-level vaccination campaigns, and results from laboratory investigation of samples and rabies suspect cases for animals. Livestock field officers and health workers collected and submitted these data as events occurred (vaccination campaigns, suspect rabid animals identified, animal bite patients reported to clinics). Researchers from Ifakara Health Institute monitored these records and followed up with users if they identified gaps without any submissions or if they received calls on a helpline indicating that difficulties had been encountered. The mobile phone-based surveillance provided relevant government stakeholders with more detailed accessible data for evaluation of the project.

The mobile phone-based system was also used to record the responses of a household survey conducted to assess initial vaccination coverage achieved and to review dog population estimates. A minimum of 30 households were sampled per village within 6 randomly selected villages for each district. Following campaigns conducted from 2013 onward, post-vaccination transects were completed by trained enumerators in each village. Transects were walked in every village on the day after the campaign in that village (~2 h duration) recording collared dogs (vaccinated) and dogs without collars (unvaccinated). Further details of these transects are provided in Sambo et al. (this issue).

In eight mainland districts in Southern Tanzania and on Pemba Island, contact tracing methods were followed to investigate declines in the numbers of bite patients. Contact tracing involved compiling bite records from mobile phone-based surveillance, and visiting households to interview bite patients and identify the status of the biting animal based on clinical criteria and circumstances of the bite. All subsequent and previous exposures and suspect rabies cases were also traced following established methods (15).

Here, we compile these data, describe progress, and discuss challenges and successes of the project, including lessons for scaling up large-scale rabies control and prevention activities elsewhere.

## Results

### Mass Vaccination of Domestic Dogs

**Figure 2:**
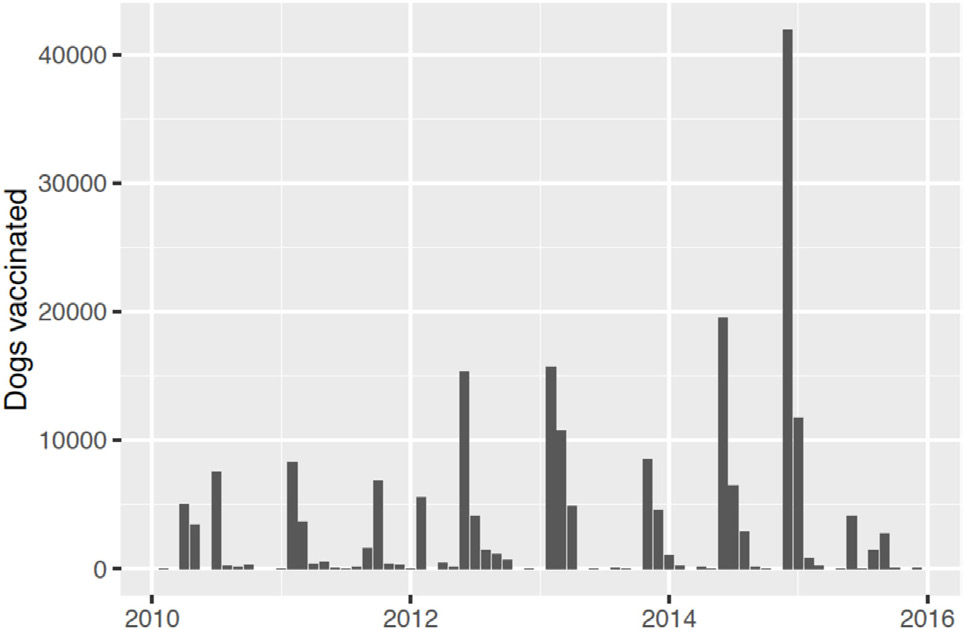
**Numbers of dogs vaccinated by month during each year of the project**.

**Figure 3:**
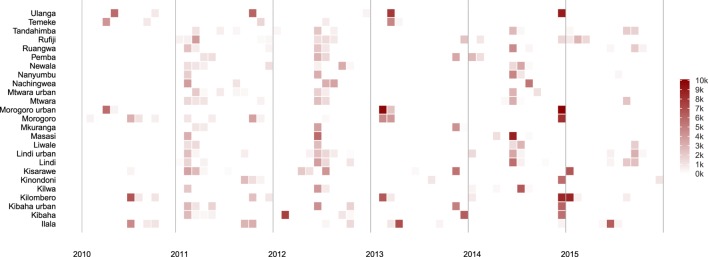
**Dogs vaccinated in each district by month during each year of the project**.

When the project was officially launched in Tanzania in 2010, mass dog vaccination campaigns were planned and delivered only in urban centers in Dar es Salaam and Morogoro as well as Ulanga and Kilombero districts where vaccination campaigns were ongoing in collaboration with the Ifakara Health Institute (Figures 2 and 3). However, operations were rapidly scaled up with mass vaccinations conducted across the rural areas of the project area in 2011. This was the first time that such large-scale mass dog vaccination campaigns had been conducted against rabies in Tanzania. The country in general, and project areas in particular, had no prior experiences with controlling rabies at this level or scale.

Relatively low numbers of dogs were vaccinated in some rural areas in the first phase of implementation and not all district-level campaigns included every village; therefore, gaps in coverage were evident. Transects conducted from 2013 until 2015 indicated an average of 65% coverage in villages where campaigns were conducted (detailed analyses of these data are underway). Coverage was probably lower than this as transects tend to miss young puppies and campaigns were not completed in every village; however, given that substantially more dogs (and villages) were vaccinated than during the initial campaigns, this represented a major improvement in implementation.

**Figure 4:**
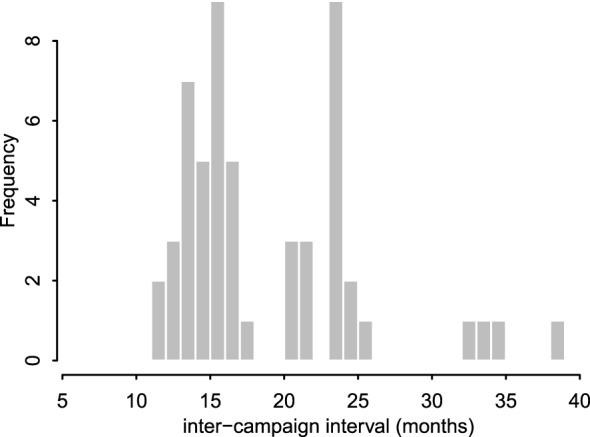
**District-level inter-campaign intervals between mass dog vaccination campaigns**. Campaigns were initially intended to occur in every district 12 months apart. However, the actual distribution of intervals between consecutive campaigns varied from 11 months to over 30 months for some intervals in districts which only completed three campaigns within the 60-month period of study (2011–2015).

Lessons learned in these early mass dog vaccination campaigns were incorporated into subsequent campaigns leading to an increase in dogs vaccinated as time went on (Figure 2). However, probably the single largest challenge to implementation was ensuring procurement and distribution of dog rabies vaccine within government and international systems. The initial plan was that dog vaccinations be conducted in each district annually. In practice, procurement challenges led to vaccination campaigns being conducted in phases involving a subset of local government authorities (Figures 2 and 3) and sometimes with long intervals between campaigns (Figure 4).

Logistical challenges included personnel changes in the WHO country office (16) and changes in vaccination procurement systems. A project officer was replaced, but recruitment was not immediate. With the absence of a project officer, several processes took longer to be implemented. Before the new procurement system, vaccines were procured internationally by the international project coordinator at the WHO headquarters, who then arranged shipment to the WHO country office in Tanzania. With the new system, all purchases related to government projects were required to go through a procurement process involving several procedures such as competitive tendering, deliberations by the procurement teams and selection of the supplier. These procedures meant that the purchase of vaccines was no longer straightforward, which led to disruptions in the vaccination schedule of the project. Lengthy intervals between campaigns were not ideal (Figures 3 and 4) and likely reduced their effectiveness. Detailed analyses of these data are underway to assess the impacts of these vaccinations and how this was affected by the disrupted schedule. A study to estimate the cost-effectiveness of the program in Tanzania found that the cost-per-dog vaccinated ranged from USD 2.5 to 22.49 across districts and phases with the average cost per phase falling from USD 11.27 in the first phase to USD 7.3 in the third phase (13). In comparison to other rabies elimination demonstration sites of KwaZulu-Natal and Cebu in the Philippines (17, 18), Tanzania had the highest cost-per-dog vaccinated mainly because of the over purchase of the vaccine in the early phases of the program and purchasing equipment for a program starting from scratch.

### Suspected Rabies Incidence, Bite Exposures, PEP Use, and Human Rabies Deaths

From January 2011 until December 2015, there were over 23,800 patient visits to clinics in the project area due to animal bites, with corresponding use of 22,295 doses of PEP. The age distribution of reported bite patients was consistent with previous findings from Tanzania and elsewhere, with younger people bitten more than adults (19–21). Overall, 45% of the bitten population was aged between 0 and 15 years of age, while over 50% of the bitten population was less than 20 years old.

**Figure 5:**
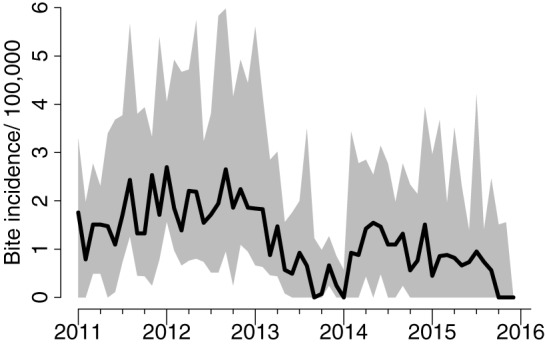
**Average bite incidence/100,000 persons per month in the project area across districts and interquartile range of district-level bite incidence**.

**Figure 6:**
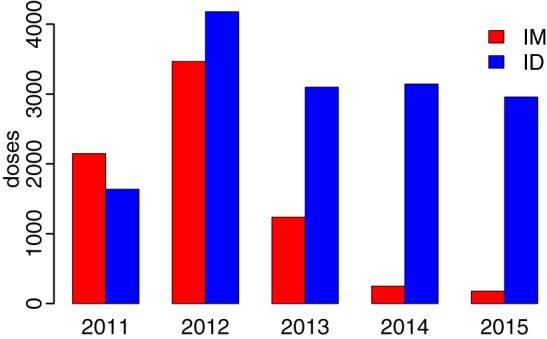
**Transition from intramuscular (IM) to intradermal (ID) administration of post-exposure prophylaxis following training of health workers in 2010**.

The number of reported bite patients increased from 2011 to 2012 from just over 1,600 bite patients to more than 2,700 (Figure 5). From 2012 onward, bites declined, but fluctuations occurred particularly in 2014. When the project started in 2010, PEP was being administered solely through the intramuscular (IM) route, which has a maximum of five doses. However, following training of health workers, a rapid shift of delivery from IM to ID—which has a maximum of four doses—was observed. The average number of PEP doses received per bite patient was 2.4. The overall reduction in bite patients led to markedly less use of PEP. The concurrent transition to ID administration (Figure 6) means that less vaccine was needed for the same number compared to IM administration. The main issue encountered during the transition related to procurement and distribution of appropriate syringes. However, even with the shift to ID administration, shortages of PEP occurred, with Ulanga and Kilombero districts in Morogoro region the worst affected. Although training in RIG and its supply from external sources were also provided, RIG was used only infrequently (<1.5% of patients) due to scarcity, and most patients given RIG were treated between 2011 and 2013.

**Figure 7:**
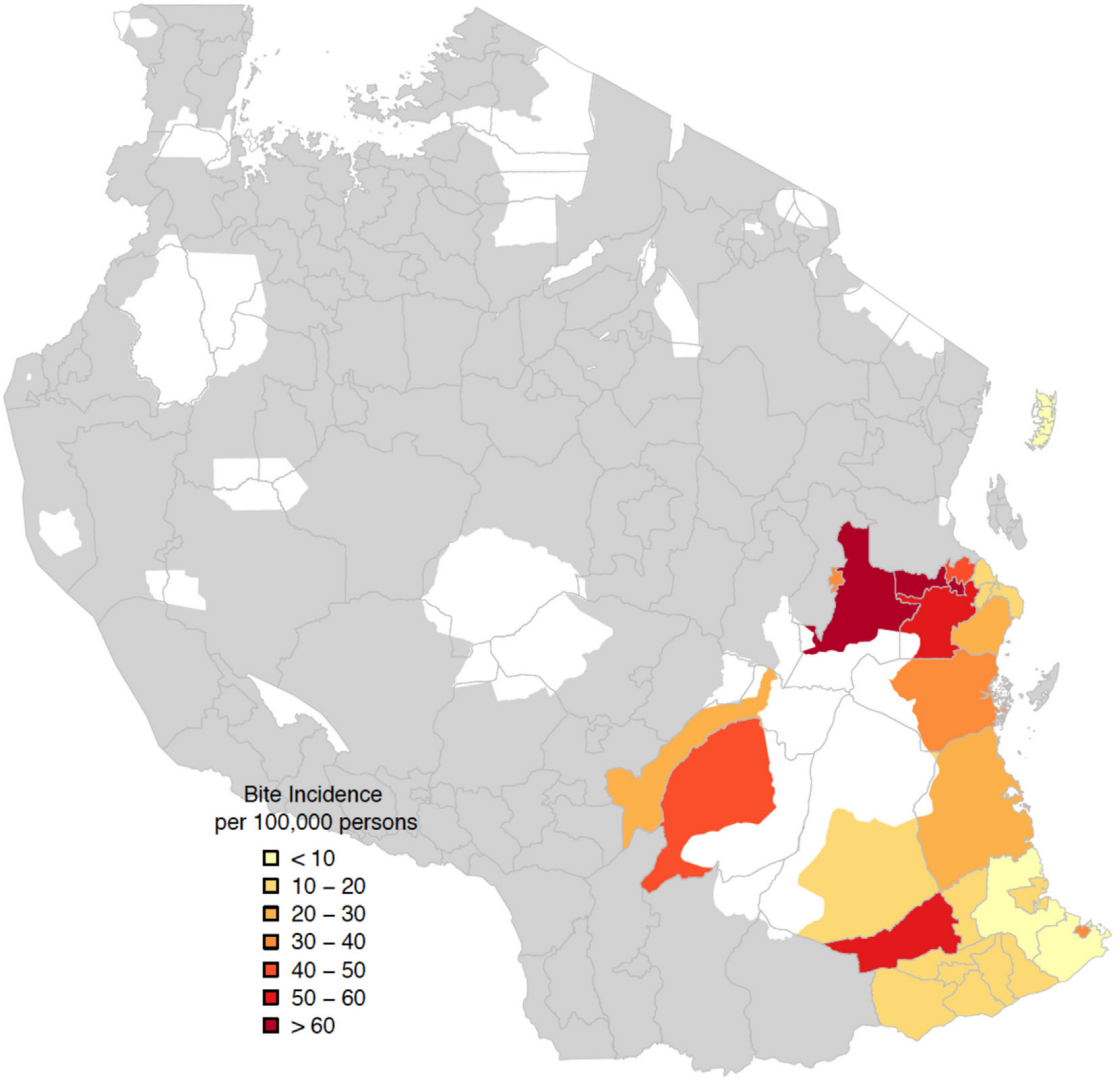
**Spatial distribution of average annual bite incidence/100,000 persons in districts in Southern Tanzania**. White zones correspond to national parks.

**Figure 8:**
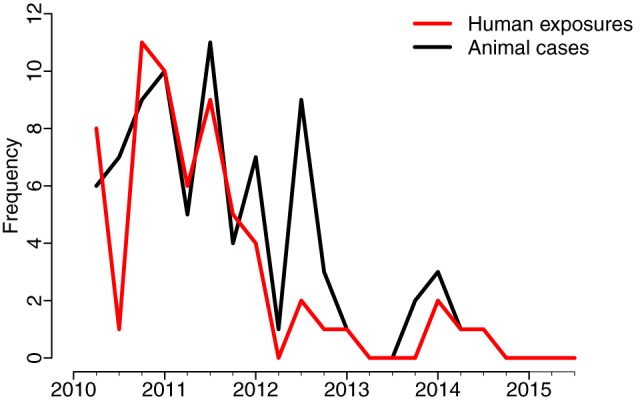
**Suspect rabies cases in animals and human exposures on Pemba Island by quarter (exposure, in the context of rabies, refers to being bitten by a possibly rabid dog)**.

The areas with the largest dog populations (Morogoro region in central Tanzania) had the highest bite incidence and demand for PEP, whereas the most notable declines in bites were observed in the most southerly districts in Lindi and Mtwara regions (Figure 7). A cost-effectiveness study estimated that the cost per human PEP administered was approximately $22.41, while the cost per life saved ranged on average from $862 to $7,859 (13). Detailed contact tracing of suspect rabies cases from bite patient records indicated that rabies was locally eliminated on Pemba island, falling from 42 suspect animal rabies cases based on clinical criteria before vaccination campaigns in 2011 to just 2 in 2014 (Figure 8). No suspect rabies cases were identified on Pemba since May 2014 despite follow-up of all bite patients, until a recent incursion was detected in August 2016. Contact tracing in the southernmost districts of Tanzania also indicated major declines in suspect rabies cases.

Prior to the demonstration project, very little data on human deaths due to rabies were available. With the project implementation, more systematic follow-up of suspected human rabies cases was practiced throughout the project areas; however, none were laboratory confirmed. In 2010, 17 deaths due to rabies were recorded in the project areas. In the following year of 2011, there were 11 recorded deaths, which further declined to 3 in 2012. There were no suspect rabies deaths recorded in 2013. However, in years 2014 and 2015, the number spiked to 4 suspect rabies deaths for both years and just 2 in 2016. This decline was likely due to mass dog vaccinations reducing incidence in dogs, while awareness campaigns encouraged increased health seeking by dog bite victims and PEP accessibility improved. Among the recorded human rabies deaths, some bite victims did not seek treatment until symptom onset, whereas others sought care, but were unable to obtain vaccines.

**Figure 9:**
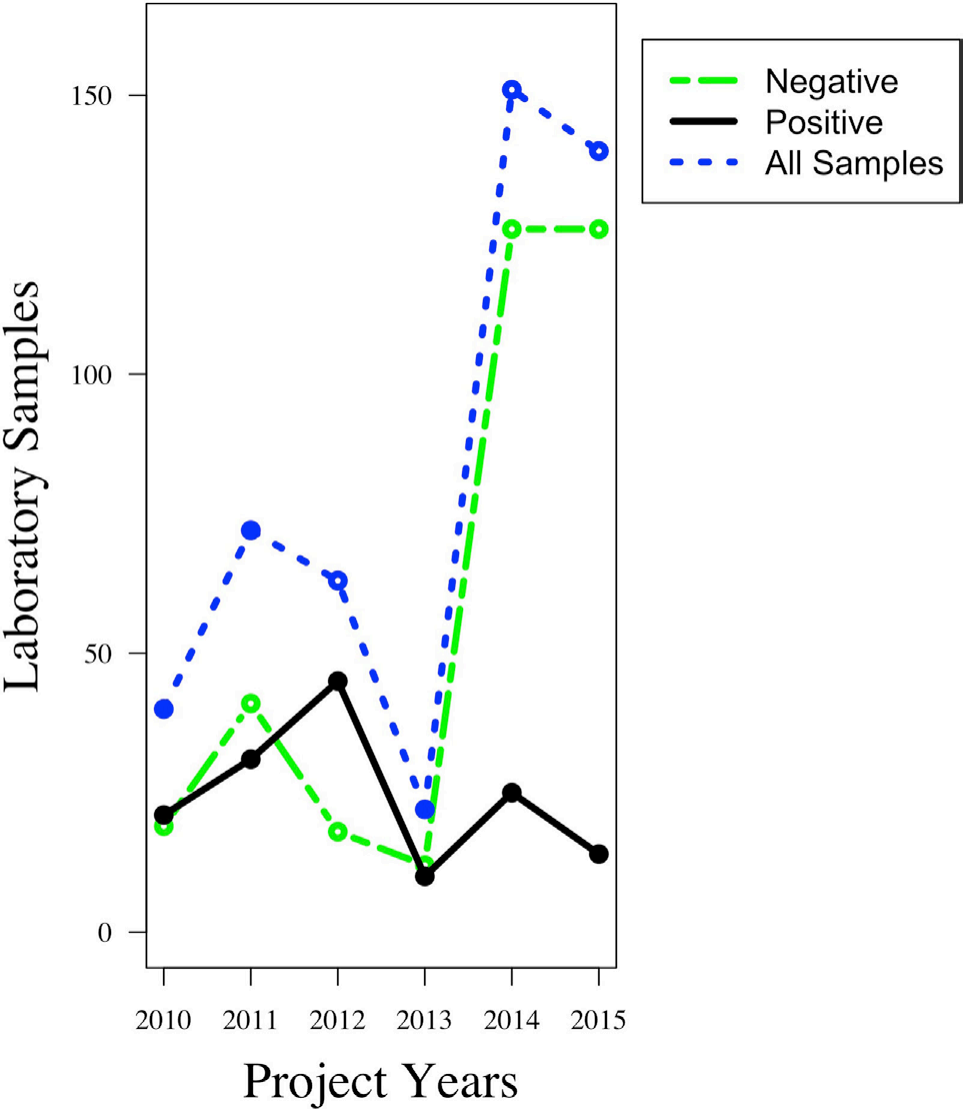
**Trends in the number of animal samples collected and analyzed between years 2010 and 2015**. Before the project begun, only a handful of samples were collected and analyzed. That number rose steadily with the full installation of the project infrastructure, and in subsequent years, the proportion of rabies-positive samples showed a decline.

Despite the training and materials for sample collection and diagnosis provided by the project, relatively few samples were submitted for testing. Local livestock officers reported considerable logistical difficulties in sample collection, including lack of resources and materials. Logistical disruptions to the project were known to have influenced sample collection during 2012 and 2013. However, despite these challenges and in comparison to the situation before the project started, more samples were collected and analyzed during the course of this project. Almost 98% of samples collected—submitted as heads of dead animals—came from domestic dogs, with the remaining 2% from cats, cows, goats, hyenas, jackals, and mongoose. The total number of samples per year showed an increasing trend with a few disruptions; there were a total of 22 samples in 2010, 40 samples in 2011, 60 samples in 2012, 22 samples in 2013, 150 samples in 2014, and 140 samples in 2015, making an average of 72 samples per year. About a quarter of all samples were diagnosed at the SUA laboratory, while three-quarters were diagnosed at the TVLA laboratory. While the number of samples submitted for diagnosis increased over the project duration, the proportion of rabies-positive samples decreased over time (Figure 9). Further analysis is underway to determine whether this fall in the number of rabies-positive samples was a direct effect of mass dog vaccination campaigns.

### Impacts on Policy

The Rabies Elimination Demonstration Project in Tanzania brought about major changes in policy. The heightened awareness of rabies among policy makers led to the establishment of offices as well as guidelines focusing on rabies elimination. For the first time, a One Health Coordination Unit was formed in the Prime Minister’s Office to coordinate health interventions of diseases and conditions whose management requires a multisectoral approach, such as rabies. In addition, a multisectoral body of experts coming from universities, ministries, research institutions, and the private sector finalized a National Rabies Control Strategy which envisions elimination of rabies in both humans and dogs in Tanzania by 2030 and takes many lessons from this demonstration project (22). To achieve the vision of elimination of rabies in Tanzania by 2030, the National Rabies Control Strategy follows the Stepwise Approach towards Rabies Elimination (SARE) (10).

## Discussion

Tanzania was among the three countries globally implementing the Rabies Elimination Demonstration Project through financial and technical assistance from the Bill and Melinda Gates Foundation (BMGF) and WHO. Activities were implemented through the MALF and the MoHCDGEC. The 5-year project aimed at controlling and eliminating rabies in domestic dogs, thereby improving surveillance and diagnostics and providing targeted delivery of PEP to rabies-exposed patients. Five regions in Southern Tanzania including Pemba Island benefited from this project. Pemba Island was included for comparison of canine rabies elimination dynamics in island and inland settings.

The Rabies Elimination Demonstration Project in Southern Tanzania has achieved major successes. Since the project inception, major declines in dog bites and suspect rabies cases have been observed, with a concomitant fall in the demand for PEP, and a shift to more cost-effective ID administration of PEP. However, rabies is yet to be eliminated entirely from the project area. Moreover, the project encountered many challenges, mainly logistical from which lessons must be learnt for the future successful elimination of rabies from Tanzania and from across sub-Saharan Africa.

This was the first large-scale One Health project in the country, with major infrastructure and human resource challenges. These included unreliable estimates of the dog population needed to purchase vaccines and consumables, shortcomings in governance capacity that hindered coordination and implementation between and within animal and human health agencies, and a lack of experience in planning and delivering dog vaccination campaigns and in routine data collection to monitor their implementation. Lack of experience was rapidly overcome as livestock field officers and health workers became familiar with project activities and were supported by follow-up provided through the surveillance system. However the structural hurdles, such as unresponsive and overly bureaucratic procurement and distribution systems, and a lack of intersectoral coordination mechanisms were more problematic. These must be critically addressed for the future success of One Health programs in sub-Saharan Africa.

Difficulties were encountered in ensuring that budgets assigned to districts were not diverted to other local competing priorities and therefore those activities were conducted as required and scheduled, given the decentralized government structure. This was most apparent in the timing of vaccination campaigns, which were not conducted annually as initially planned. Interruption in campaign implementation is a recurring issue in LMICs, with problems encountered in the other project demonstration sites (23), but less than in Tanzania. An important question to address in future is therefore how such interruptions impact on progress toward rabies elimination and how these can be minimized.

Implications of overestimating the dog population in the project proposal were quite important, especially as Tanzania was allocated a large proportion of the total BMGF 5-year funding for rabies pilot projects. Its impact on the cost-effectiveness of the program is obvious considering that the actual dog population to be immunized was about a third of the initially estimated target population size. As a consequence, larger than needed quantities of dog vaccines were procured and stored for long periods of time. Moreover, funds for dog vaccination campaigns were kept unused with the possibility of being reallocated to more pressing activities, though for a large part excess dog vaccines (and budget) were available for future years. Subsequent vaccination campaigns and post-vaccination monitoring enabled assessment of coverage and informed future vaccine purchase (21). However, procurement obstacles for both vaccines and consumables were considerable. Partly, this was due to a lack of continuity in project leadership and shortcomings in organizational capacity (16). These challenges highlight the catalytic role that mechanisms such as the OIE rabies vaccine bank can play in overcoming bureaucratic challenges such as the tendering process to ensure a reliable supply of low-cost, high-quality vaccines.

Similar arguments apply to the supply of lifesaving PEP, which could be transformed if rabies post-exposure vaccines were incorporated within the portfolio of vaccines supported by the GAVI, the Vaccine Alliance. ID administration of PEP is cheaper than IM (reducing costs by at least 60% due to savings in vaccine) and equally immunogenic (12, 24–27). The rapid transition to ID administration demonstrated that health workers were capable of implementing this more cost-effective vaccine-sparing approach. Provision of free PEP administered intradermally to bite victims attending local health facilities greatly improved the PEP access across project areas. Nonetheless, even with this improved provision, PEP shortages still occurred, even at district hospitals, and this continues to be a challenge across the country more generally. Improved approaches are therefore needed for the distribution of lifesaving human rabies vaccines, given the unpredictability of demand, potential for epidemics, and urgency with which they must be administered.

Critically, many lessons spanning management, logistics, organization, implementation, and technological areas were learned through this project, which should be applied to the roll out of rabies elimination programs elsewhere in Tanzania and sub-Saharan Africa. These include the need to have a dedicated management unit with focused responsibilities to deal with rabies, for close cooperation of key sectors and stakeholders involved, and for flexible and responsive procurement and distribution systems. The Tanzanian government recognized the need for intersectoral financing and coordinating mechanisms and recently established the One Health Coordination Unit under the Prime Minister’s Office to deal with endemic and emerging epidemics (28). Major costs for implementing the program were attributed to *per diem* payments for livestock field officers conducting campaigns out of their offices (13). This has prompted investigations into more efficient delivery methods for mass dog vaccination campaigns. The use of innovative methods such as mobile phones to support comprehensive surveillance infrastructure with timely data collection also improved decision-making. Spatial visualization of surveillance data and feedback from frontline health workers and livestock field officers captured through the mobile phone-based system were used to communicate to policy makers the role of local government and the differences in implementation and results across the project area (14).

Overall, it is clear that the program delivered valuable public health benefits, but it is also evident that if dog vaccinations are not continued these gains will be lost. The recent incursion on Pemba highlights this situation, where no vaccination campaigns were conducted since early 2014. However, the improved surveillance resulted in early detection and prompted a strong outbreak response. Moreover, it appears as though there is now only limited circulation of rabies in the southernmost districts in Tanzania and that elimination across a wider region is within reach. The ultimate success of the project will be seen in whether the sustainability plan for continued mass dog vaccinations is achieved and whether this leads to rabies elimination across the project area and spurs progress toward the elimination of rabies nationally.

## Ethics Statement

This was a demonstration project that involved mass dog vaccination campaigns. However, aspects of mobile phone surveillance, since they involved humans, were granted ethical clearance by the Institutional Review Board of Ifakara Health Institute and the Medical Research Coordinating Committee of the National Institute for Medical Research of Tanzania (NIMR/HQ/R.8a/Vol.IX/946).

## Author Contributions

EAM conceived the general structure of the paper, analyzed data, and wrote the manuscript; TL contributed in writing the manuscript; SM and SL contributed by providing information about logistic challenges of the program; MM contributed by providing information about program implementation and challenges; CN contributed by providing information related to diagnostics part of the program; JC contributed by providing information about the implementation of the mobile phone surveillance; ZM contributed by providing information about the mobile phone surveillance; MS contributed information about estimation of human:dog ratio and program implementation; GM contributed information about implementation from the health sector; KL, SM, LS, GJ, CN, JC, ZM and MS all contributed to data collection and processing; KR, RM and ST contributed through data analysis and graphics; KL contributed information about project implementation; AN contributed information about project implementation from the WHO side; F-XM provided information about the early stages of the program; BA-R contributed information about project implementation and logistics; GJ contributed information about project implementation’s challenges and success; EMM contributed ministry-specific information; and RK, TL, SC, and KH made substantial contributions to the conception of design, data analysis, and general layout of the work. RM contributed in reviewing and proof-reading the manuscript; LS contributed in field-specific information; ST contributed in project challenges-specific information.

## Conflict of Interest Statement

The authors declare that the research was conducted in the absence of any commercial or financial relationships that could be construed as a potential conflict of interest.

## References

[1] Garg SR Rabies in Man and Animals. New Delhi: Springer (2014).

[2] WHO. WHO Expert Consultation on Rabies: Second Report. Geneva: World Health Organization (2013). 24069724

[3] Nel LH Taylor LH Balaram D Doyle KA Global partnerships are critical to advance the control of neglected zoonotic diseases: the case of the global alliance for rabies control. Acta Trop (2015) 165:274–9. 10.1016/j.actatropica.2015.10.014 26519885

[4] Hampson K Coudeville L Lembo T Sambo M Kieffer A Attlan M Estimating the global burden of endemic canine rabies. PLoS Negl Trop Dis (2015) 9(4):e0003709 10.1371/journal.pntd.0003709 25881058 PMC4400070

[5] Knobel DL Cleaveland S Coleman PG Fèvre EM Meltzer MI Miranda MEG Re-evaluating the burden of rabies in Africa and Asia. Bull World Health Organ (2005) 83(5):360–8. 15976877 PMC2626230

[6] Lembo T Hampson K Kaare MT Ernest E Knobel D Kazwala RR The feasibility of canine rabies elimination in Africa: dispelling doubts with data. PLoS Negl Trop Dis (2010) 4(2):e626. 10.1371/journal.pntd.0000626 20186330 PMC2826407

[7] Kaare M Lembo T Hampson K Ernest E Estes A Mentzel C Rabies control in rural Africa: evaluating strategies for effective domestic dog vaccination. Vaccine (2009) 27(1):152–60. 10.1016/j.vaccine.2008.09.054 18848595 PMC3272409

[8] Zinsstag J Schelling E Roth F Bonfoh B de Savigny D Tanner M. Human benefits of animal interventions for zoonosis control. Emerg Infect Dis (2007) 13(4):527–31. 10.3201/eid1304.060381 17553265 PMC2725951

[9] Zinsstag J Dürr S Penny MA Mindekem R Roth F Gonzalez SM Transmission dynamics and economics of rabies control in dogs and humans in an African city. Proc Natl Acad Sci U S A (2009) 106(35):14996–5001. 10.1073/pnas.0904740106 19706492 PMC2728111

[10] Taylor L Developing a stepwise approach for rabies prevention and control. Proceedings FAO/GARC Workshop Rome (2014).

[11] Knobel DL Laurenson MK Kazwala RR Boden LA Cleaveland S. A cross-sectional study of factors associated with dog ownership in Tanzania. BMC Vet Res (2008) 4(1):1. 10.1186/1746-6148-4-5 18230137 PMC2262882

[12] Ambrozaitis A Laiškonis A Balčiuniene L Banzhoff A Malerczyk C. Rabies post-exposure prophylaxis vaccination with purified chick embryo cell vaccine (PCECV) and purified Vero cell rabies vaccine (PVRV) in a four-site intradermal schedule (4-0-2-0-1-1): an immunogenic, cost-effective and practical regimen. Vaccine (2006) 24(19):4116–21. 10.1016/j.vaccine.2006.02.036 16545510

[13] Hatch B Anderson A Sambo M Maziku M Mchau G Mbunda E Towards canine rabies elimination in South-eastern Tanzania: assessment of health economic data. Transbound Emerg Dis (2016). 10.1111/tbed.12463 26916104

[14] Mtema Z Changalucha J Cleaveland S Elias M Ferguson HM Halliday JE Mobile phones as surveillance tools: implementing and evaluating a large-scale intersectoral surveillance system for rabies in Tanzania. PLoS Med (2016) 13(4):e1002002. 10.1371/journal.pmed.1002002 27070315 PMC4829224

[15] Hampson K Dushoff J Cleaveland S Haydon DT Kaare M Packer C Transmission dynamics and prospects for the elimination of canine rabies. PLoS Biol (2009) 7(3):e1000053. 10.1371/journal.pbio.1000053 19278295 PMC2653555

[16] WHO. Report of the Fourth Meeting of the International Coordinating Group of the Bill & Melinda Gates Foundation–World Health Organization Project on Eliminating Human and Dog Rabies in Cebu City, Philippines, 2–4 October 2012. Geneva (2013). Available from: http://apps.who.int/iris/bitstream/10665/79216/1/WHO_HTM_NTD_NZD_2013.1_eng.pdf

[17] Miranda L Miranda M Hatch B Deray R Shwiff S Roces M Towards canine rabies elimination in Cebu, Philippines: assessment of health economic data. Transbound Emerg Dis (2015) 64(1):121–9. 10.1111/tbed.12350 25885005

[18] Shwiff S Hatch B Anderson A Nel L Leroux K Stewart D Towards canine rabies elimination in KwaZulu-Natal, South Africa: assessment of health economic data. Transbound Emerg Dis (2014) 63(4):408–15. 10.1111/tbed.12283 25414096

[19] Cleaveland S Fevre EM Kaare M Coleman PG. Estimating human rabies mortality in the United Republic of Tanzania from dog bite injuries. Bull World Health Organ (2002) 80(4):304–10. 12075367 PMC2567765

[20] Hampson K Dobson A Kaare M Dushoff J Magoto M Sindoya E Rabies exposures, post-exposure prophylaxis and deaths in a region of endemic canine rabies. PLoS Negl Trop Dis (2008) 2(11):e339. 10.1371/journal.pntd.0000339 19030223 PMC2582685

[21] Sambo M Cleaveland S Ferguson H Lembo T Simon C Urassa H The burden of rabies in Tanzania and its impact on local communities. PLoS Negl Trop Dis (2013) 7(11):e2510. 10.1371/journal.pntd.0002510 24244767 PMC3820724

[22] United Republic of Tanzania. National Rabies Control Strategy. Dar es Salaam: United Republic of Tanzania through the Ministry of Agriculture Livestock and Fisheries (MALF) (2017).

[23] WHO. Report of the Fifth Meeting of the International Coordinating Group of the World Health Organization and the Bill & Melinda Gates Foundation Project on Eliminating Human and Dog Rabies in Dar es Salaam, United Republic of Tanzania, 8–10 October 2013. Geneva (2014). Available from: http://apps.who.int/iris/bitstream/10665/102317/1/WHO_HTM_NTD_NZD_2014.2_eng.pdf?ua=1

[24] Hampson K Cleaveland S Briggs D. Evaluation of cost-effective strategies for rabies post-exposure vaccination in low-income countries. PLoS Negl Trop Dis (2011) 5(3):e982. 10.1371/journal.pntd.0000982 21408121 PMC3050908

[25] Khawplod P Wilde H Tepsumethanon S Limusanno S Tantawichien T Chomchey P Prospective immunogenicity study of multiple intradermal injections of rabies vaccine in an effort to obtain an early immune response without the use of immunoglobulin. Clin Infect Dis (2002) 35(12):1562–5. 10.1086/344954 12471579

[26] Suntharasamai P Warrell M Viravan C Chanthavanich P Looareesuwan S Supapochana A Purified chick embryo cell rabies vaccine: economical multisite intradermal regimen for post-exposure prophylaxis. Epidemiol Infect (1987) 99(03):755–65. 3428378 10.1017/s0950268800066619 PMC2249234

[27] WHO. Report of a WHO Consultation on Intradermal Applications of Human Rabies Vaccines, Geneva, 13–14 March 1995. Geneva: WHO (1995).

[28] Afrique One Alliance. Control of Epidemic Disease in Tanzania: Dr Sayoki Mfinanga Presented the Contribution of Afrique One-ASPIRE. (2016). Available from: http://afriqueoneaspire.org/activities/control-of-epidemic-disease-in-tanzania-dr-sayoki-mfinanga-presented-the-contribution-of-afrique-one-aspire/

